# Effect of *Azolla pinnata* inclusion in corn silage on digestibility, ruminal fermentation, blood parameters and growth performance of Barki male lambs

**DOI:** 10.1007/s11250-025-04633-y

**Published:** 2025-09-18

**Authors:** Usama A. Nayel, Gamal A. Baraghit, Mohamed Y. Elaref, Mohamed A. Abd-Elhakeem, Eman I. Saddick

**Affiliations:** 1 Department of Animal Production, Faculty of Agriculture, Menoufia University, Shibin el Kom, Egypt; 2 Department of Animal Production, Faculty of Agriculture, Sohag University, Sohag, 82524 Egypt

**Keywords:** Azolla-corn silage, Silage characteristics, Digestibility, Rumen fermentation, Lambs’ growth

## Abstract

Azolla (*Azolla pinnata*- Ap) is a novel and unconventional source of animal feed, which enhances the nutritional value and reduces feeding costs. Azolla has not been used in animal feeding as silage before, so the aim of this study was to investigate the impact of increasing levels of Azolla as a partial component in corn silage on the nutrient digestibility, rumen fermentation, blood parameters, and growth performance of Barki male lambs. In ensilage processes, different levels of wilted Azolla were mixed in chopped corn plants (without cobs) in four treatments, as follows: (1) 100% chopped corn without Azolla; (2) 95% chopped corn plus 5% Azolla; (3) 90% chopped corn plus 10% Azolla; (4) 85% chopped corn plus 15% Azolla. A growth trial was conducted using twenty-eight healthy Barki male lambs (8 months old, average initial body weight of 31.25 ± 0.60 kg), which were divided into four groups (*n* = 7). Lambs in each group were fed one of the four types of silage produced as a single diet at 3% dry matter of body weight. Results revealed that the inclusion of Azolla in corn silage significantly (*P*˂0.05) increased crude protein content and enhanced physical and fermentation characteristics. All nutrients digestibility were improved in corn silage mixed with different levels of Azolla compared to corn silage (*P*˂0.05). The concentrations of ammonia and total volatile fatty acid in the rumen reached their highest values at 3 h post-feeding. They were significantly higher (*P* = 0.004 and *P* = 0.045, respectively) for lamb groups fed any of three varieties of Azolla-corn silage than lambs fed corn silage. The highest (*P*˂0.05) values of body weight at 60 and 90 day, total body gain and average daily gain were recorded in lambs fed silage contained 15% Azolla, followed by those fed silage contained 10% and 5% Azolla, while the lowest values were recorded in lambs fed control silage. Lambs fed silage contained 10% and 10% Azolla had the greatest total protein and albumin values (*P* = 0.027 and *P* = 0.021, respectively), followed by lambs fed silage contained 5% Azolla, while those fed corn silage had the lowest values. In conclusion, the physical, chemical and fermentation characteristics of silage of Azolla corn mixture were enhanced. They can be used successfully in Barki male lamb rations, with beneficial effects on digestibility, nutritive value, rumen fermentation and growth performance.

## Introduction

In Egypt, animal feed that satisfies nutritional requirements lacks quantity and quality (Elaref et al. 2020). High-quality and balanced ruminant rations necessitate protein-rich sources, which are costly (Abdullah et al. 2024). It is necessary to intensify efforts to find novel and unconventional sources of animal feed that enhance their nutritional value and lower the cost of rations (Abo-Donia and Nayel. 2022; Abo-Donia et al. 2022; Kewan et al. 2021; Ali et al. 2015). Given the growing interest in preserving the environment and the need to deploy renewable and sustainable resources, interest has increased in the multiple uses of the Azolla plant, not only as animal feed but also as a human food, medicine, biofertilizer on a variety of crops, and a water purifier. In addition, it has been used to produce hydrogen fuel, biogas, control weeds, and reduce the volatilization of ammonia that accompanies chemical nitrogen fertilizers (Wagner 1997).

Azolla (*Azolla pinnata*) is an aquatic fern that floats in the water (Abd El-Ghany 2020; Mooventhan et al. 2019) and is widely distributed throughout the world in the tropics, subtropics, and warm temperate regions (Costa et al. 2009; Parris 2001). Pillai et al. (2004) reported that Azolla grows rapidly and produces 730 t/ha of fresh matter annually, containing 56 and 20 t/ha of dry matter and protein content, respectively. Azolla is a rich source of high-quality protein and contains essential amino acids, vitamins, beta-carotene, minerals, and many biopolymers (Kumar and Chander 2017; Joysowal et al. 2018).

Azolla’s rapid growth and high nutritional value have increased interest in its use as a non-traditional feed for livestock, poultry, and fish (Varun Singh et al. 2021). Indira et al. (2009) state that the DM digestibility, daily body gain, and feed efficiency of Nellore sheep or buffalo improve by replacing 30 or 50% of the protein in their diet. Sharma et al. (2021) concluded that supplementing 250 g of green Azolla to concentrate diet improves the average daily gain and feed conversion ratio in male kids by 29.34% and 17.38%, respectively, compared with no Azolla supplement. In addition, some previous studies indicated that rumen fermentation parameters were improved by including Azolla in animal feeds; the NH3-N concentration increased (Abou El-Fadel et al. 2020; Kumar et al. 2015b); and the concentration of TVFA increased (Hassanein et al. 2023; Sharma et al. 2021). In animals and birds, Azolla helps with weight gain and the production of milk and eggs (Herath et al. 2023). Nasir et al. (2022) suggest Azolla as a favorable alternative for animal feed components that contribute to providing a healthy food and environmental system.

Previous studies have examined Azolla plants (fresh or dried) as an alternative low-cost source of protein and their impact on livestock productivity. A detailed examination of earlier research reveals a lack of knowledge on using Azolla in silage production and how it affects ruminant performance. Therefore, the present study aimed to investigate the effects of including the Azolla plant as a partial component of corn silage on the nutrient digestibility, rumen fermentation, blood parameters, and growth performance of Barki lambs.

## Materials and methods

The current study was conducted at the Animal Production Department of the Faculty of Agriculture, Menoufia University, under the Scientific Research Ethics and Animal Use Committee (SRE & AUC) at Menoufia University, Faculty of Agriculture (Reference No: 013-SRE&AUC-MUAGR-04-2024).

### Experimental design, animals and diets

#### Silage perpetration and evolution

In the current study, a silage manufacture and feeding experiment was conducted to investigate the impact of increasing levels of Azolla (*Azolla pinnata*- Ap*)* in corn silage on nutrient digestibility, rumen fermentation, blood parameters and growth performance of Barki male lambs. Fresh corn plants (without cobs) at 90 days old and with 65% moisture content (milk stage) were obtained from a commercial farm in the Menoufia governorate and chopped into 2–3 cm in length. Fresh Azolla was collected at 17 days of age and wilted in the air till it reached 70% moisture content. The different levels of wilted Azolla were mixed in chopped corn plants (without cobs) according to the following four treatments at ensilage processes, respectively: (1) consisted of 100% chopped corn without Azolla (Con); (2) contained a mixture of 95% chopped corn plus 5% Azolla (5Ap); (3) contained a mixture of 90% chopped corn plus 10% Azolla (10Ap); (4) contained a mixture of 85% chopped corn plus 15% Azolla (15Ap). Tight plastic barrels were used for ensiling. Molasses were diluted with water (1:1) and sprinkled on plant materials at a rate of 5%. The prepared material was compressed as much as possible using a vacuum sealer to exclude excess air and the barrels were filled to the top and then tightly sealed with lids. Heavy weights were placed on top of the sealed barrels, stored at room temperature (25 °C), and left for 45 days (ensiling period). The chemical composition of the four silage treatments before ensiling is shown in Table 1. At the end of ensiling period, Samples were taken from the four silage treatments for physical (pH, DM content and Fleig score), chemical composition (OM, CP, CF, EE, NFE and Ash %) and fermentation characteristics (lactic and acetic acid content, according to Madrid et al. (1999) by HPLC methods).

**Table 1:** Chemical composition of the four silage treatments (before ensiling)

Chemical composition, %	Experimental diets			
	Con	5Ap	10Ap	15Ap
DM	32.21	32.04	31.71	31.21
OM	91.58	90.87	90.77	90.03
CP	8.51	8.93	9.66	9.81
CF	24.61	23.78	23.80	23.13
EE	3.34	3.95	4.31	4.04
NFE	55.12	54.21	53.00	53.05
Ash	8.42	9.13	9.48	9.97

Con: silage of corn (100% fresh corn without *Azolla pinnata***);** 5Ap, mixture of (95% fresh corn + 5% *Azolla pinnata*); 10Ap, mixture of (90% fresh corn + 10% *Azolla pinnata*); 15Ap, mixture of (85% fresh corn + 15% *Azolla pinnata*); DM, dry matter; OM, organic matter; CP, crude protein; CF, crude fiber; EE, ether extract; NFE, nitrogen-free extract

#### Growth performance

A completely randomized design was followed in the growth trial using twenty-eight healthy Barki male lambs (8 months old, average initial body weight of 31.25 ± 0.60 kg), which were divided into four homogenous groups (seven lambs/ group) according to their live body weight. Animals in each group were housed together in a 3 × 3.5 m closed barn with access to fresh water 24 h a day. By the guidelines of the NRC (2007), the animals in each group were fed one of the four types of silage produced as a single diet at 3% DM of body weight to meet their nutrient requirements. Throughout the 105-day (15-day adaptation period + 90-day growth trial period), the experimental feed was offered to animals twice a day at 8:00 and 16:00. Every 10 days, the amount of feed was modified based on the changes in the lambs’ body weight. Lambs were weighted once monthly before the morning feeding until the end of the growth trial period to calculate total weight gain and average daily gain.

#### Digestibility trial

After the growth trial was finished, the digestibility experiment was initiated by keeping 16 lambs individually (four lambs from each group) with an average body weight of 42.37 ± 2.15 kg in metabolic cages for three weeks as a preliminary period followed by five consecutive days for samples collection. Samples from residual feed (about 50 g) were obtained, mixed, ground through a 2 mm mill screen, and kept to estimate the digestion coefficients of DM, OM, CP, EE, and CF. Likewise, 10% of the weight of the feces was obtained each day, dried at 70 °C for 24 h, ground through a 2 mm mill screen and stored for chemical analysis. Residual feed and feces samples were subjected to chemical analysis using the AOAC (2011). The classic formula of McDonald et al. (1995) was used to calculate the total digestible nutrients (TDN) and digestible crude protein (DCP). Daily urine was quantitatively collected during the collection periods at 9:00 a.m. before feeding in containers containing 50 ml of 0.1 N HCl (pH < 2.00) to determine nitrogen balance. A quantity of 10% of the total urine from each ram was withdrawn and kept in glass bottles in a deep freezer (-20˚C) for later determination of N content.

#### Rumen fermentation characteristics

At the end of the digestibility trial, rumen content samples were obtained at 0, 3 and 6 h post-feeding for each animal using a rubber stomach tube to determine the rumen fermentation characteristics (*p*H, NH3-N and VFA). Samples were strained through four layers of cheese-cloth and homogenized and *p*H was immediately determined using the *p*H meter (Model HI 8424). About 0.5 ml of toluene and 1 ml of paraffin oil were added to each sample and stored in a deep freezer (-20˚C) until chemical analysis. The steam distillation method described by Eadie et al. (1967) was used to determine the volatile fatty acids (VFA) after acidification of rumen samples using concentrated ortho-phosphoric acid and 0.1 N hydrochloric acid. Free ammonia-N in the rumen samples was determined according to Conway (1978).

#### Blood sampling and blood parameters

At the end of the digestibility trial, blood samples were collected from each lamb in two tubes 2 h post-feeding via the jugular vein. The first was K-EDTA tube used to determine hematological parameters using automated veterinary hematology analyzer. The other tube was used to obtain the blood serum without anti-coagulant and centrifuged at 3500 rpm for 20 min. Serum was preserved until analysis at -20 ˚C to determine total protein, albumin, globulin, glucose, urea, creatinine, alanine transaminase (ALT), aspartate transaminase (AST), and alkaline phosphatase (ALP) by calorimetric methods using commercial kits according to the manufacturer’s guidelines.

#### Statistical analysis

All data collected from growth and digestibility trials were statistically analyzed using General Linear Models (GLM) of SAS (version 9.3.1, SAS Institute Inc., Cary, NC, USA). Differences among treatments were evaluated using the Multiple Range test, according to Duncan (1955). The statistical model used to analyze the obtained data was:

$$Y_{ij}=\mu+A_{i}+\varepsilon_{ij}$$

Where *Y*_*ij*_ is the dependent variable (nutrient digestibility, rumen fermentation characteristics growth performance, and blood parameters), *µ* is the overall mean, *A*_*i*_ is the fixed effect of Azolla level (Con, 5Ap 10Ap, and 15Ap), and *ε*_*ij*_ is the residual error.

## Results

### Silage evaluation

The chemical composition and fermentation characteristics of corn silage ensilaged with or without different levels of Azolla are shown in Table 2. The pH value and the DM content decreased (*P* = 0.001 and *P* = 0.003, respectively) when the amount of Azolla in the manufactured silage increased. The Fleig score had its highest (*P* = 0.034) value in 15Ap, followed by 5Ap and 10Ap, and the control group had the lowest value.

**Table 2:** Chemical composition and fermentation characteristics of corn ensilaged with or without Azolla

Item	Experimental diets				SEM	*P* value
	Con	5Ap	10Ap	15Ap		
*p*H	3.96^a^	3.85^b^	3.77^bc^	3.70^c^	0.03	0.001
DM (%)	31.41^a^	31.20^a^	29.87^b^	30.10^b^	0.21	0.003
Fleig score	109.52^b^	113.40^ab^	113.94^ab^	117.20^a^	0.99	0.034
Chemical composition, %						
OM (%)	88.71	88.87	89.45	89.10	0.16	0.674
CP (%)	7.60^c^	7.94^bc^	8.46^ab^	8.89^a^	0.16	0.007
CF (%)	26.10^a^	25.40^ab^	24.60^b^	25.22^ab^	0.19	0.023
EE (%)	3.97	4.23	4.60	4.68	0.12	0.797
NFE (%)	51.04	51.30	51.79	50.31	0.27	0.288
Ash (%)	11.29	11.13	10.55	10.90	0.16	0.674
Fermentation characteristics, mg/g DM						
Lactic acid	17.61^d^	20.20^b^	26.12^a^	19.73^c^	0.95	˂0.001
Acetic acid	14.83^a^	13.55^b^	13.66^b^	13.62^b^	0.16	˂0.001

Con: silage of corn (100% fresh corn without *Azolla pinnata***);** 5Ap, mixture of (95% fresh corn + 5% *Azolla pinnata*); 10Ap, mixture of (90% fresh corn + 10% *Azolla pinnata*); 15Ap, mixture of (85% fresh corn + 15% *Azolla pinnata*); Flieg score = 220 + (2 x% Dry Matter − 15) − 40 x *p*H according to Kilic (2006), DM, dry matter; OM, organic matter; CP, crude protein; CF, crude fiber; EE, ether extract; NFE, nitrogen-free extract

On a DM% basis, results in Table 2 show that increasing Azolla levels in 10Ap and 15Ap significantly (*P* = 0.007) enhanced the CP content compared to the corn silage in control and 5Ap. The protein content increased in 10Ap and 15Ap by 6.5 and 11.7%, respectively. On the contrary, increasing Azolla levels in 5Ap, 10Ap and 15Ap significantly (*P* = 0.023) reduced the CF content compared to the control. The OM, EE, NFE, and ash content were not significantly (*P* > 0.05) affected by increasing Azolla levels in corn silage.

Additionally, Table 2 showed that corn silage (con) had a lower lactic acid concentration (*P*˂0.001) than corn silage mixed with varying levels of Azolla; 10Ap silage had the greatest value, followed by 5Ap and 15Ap silage. Conversely, acetic acid concentrations in corn silage combined with varying levels of Azolla reduced (*P*˂0.001), and there were no discernible variations between them compared to corn silage.

### Nutrient digestibility

The nutrient digestibility, nutritive value and nitrogen balance of Barki male lambs fed corn silage or corn silage mixed with different levels of Azolla are presented in Table 3. Chemical analysis revealed an improvement in the digestibility coefficients of all nutrients for corn silage mixed with different levels of Azolla compared to corn silage and the highest (*P*˂0.05) nutrient digestibility coefficients were obtained in the 10Ap silage. The nutrient digestibility coefficients of 15Ap silage also improved (*P*˂0.05), except for the EE. The lowest rate of improvement in nutrient digestibility coefficients was obtained in the 5Ap silage, which did not differ significantly (*P* > 0.05) from corn silage in the control. Moreover, both 10Ap and 15Ap silage showed improvements in DCP and TDN% (*P* = 0.020 and *P* = 0.003, respectively) compared to 5Ap silage and corn silage, which did not differ significantly from each other (Table 3).

**Table 3:** Nutrient digestibility, nutritive value and nitrogen balance of Barki male lambs fed corn silage or corn silage mixed with different levels of Azolla

Item	Experimental diets				SEM	*P* value
	Con	5Ap	10Ap	15Ap		
Nutrient digestibility, %						
DM	59.82^c^	61.03^c^	70.85^a^	67.25^b^	1.41	˂0.001
CP	60.53^c^	63.38^bc^	71.65^a^	68.94^ab^	1.53	0.008
CF	60.52^b^	62.70^ab^	67.96^a^	67.35^a^	1.11	0.014
EE	69.91^ab^	73.14^a^	73.76^a^	66.06^b^	1.20	0.047
NFE	63.99^bc^	62.12^c^	72.11^a^	65.92^b^	1.22	0.001
Nutritive value, %						
DCP	4.60^b^	5.03^ab^	6.06^a^	6.12^a^	0.24	0.020
TDN	55.83^c^	55.92^c^	63.52^a^	59.37^b^	1.12	0.003
Nitrogen balance, g/day						
Intake-N	35.97	33.69	34.12	38.31	0.81	0.159
Feces-N	13.13^a^	13.27^a^	9.63^c^	11.90^b^	0.51	˂0.001
Urine-N	15.52	11.89	12.80	14.97	0.76	0.293
Balance-N	7.32^b^	8.52^b^	11.69^a^	11.44^a^	0.83	0.007

Con: silage of corn (100% fresh corn without *Azolla pinnata***);** 5Ap: mixture of (95% fresh corn + 5% *Azolla pinnata*); 10Ap: mixture of (90% fresh corn + 10% *Azolla pinnata*); 15Ap: mixture of (85% fresh corn + 15% *Azolla pinnata*); DM, dry matter; CP, crude protein; CF, crude fiber; EE, ether extract; NFE, nitrogen-free extract: DCP, digested crude protein; TDN, total digestible nutrients

The intake-N and urine-N did not differ significantly (*P* = 0.159 and *P* = 0.293, respectively) in lambs fed corn silage or corn silage mixed with different levels of Azolla (Table 3). Feces-N decreased (*P*˂0.001) significantly in lambs fed 10Ap and 15Ap silage compared to those fed corn silage or 5Ap silage. Due to significant decreases in feces-N and increases in nitrogen retained in the body, lambs fed 10Ap and 15Ap silage had a better nitrogen balance (*P* = 0.007) when compared to those fed corn silage or 5Ap silage.

### Rumen fermentation parameters

Table 4 revealed that lambs fed corn silage or corn silage mixed with different levels of Azolla at zero time (before feeding) did not affect (*P* > 0.05) the rumen pH value, VFA and NH3-N concentration. While, at three hours post-feeding, the rumen pH values were significantly (*P* = 0.021) increased in lambs fed corn silage mixed with different levels of Azolla compared with control silage, differences among the three levels of Azolla were not significant. At 6 h post-feeding, rumen pH values did not differ significantly (*P* = 0.485) among experimental lamb groups.

**Table 4:** Rumen fermentation parameters of Barki male lambs fed corn silage or corn silage mixed with different levels of Azolla

Item		Experimental diets				SEM	*P* value
		Con	5Ap	10Ap	15Ap		
pH	0 h	6.86	6.85	6.92	6.89	0.03	0.895
	3 h	6.19^b^	6.72^a^	6.83^a^	6.77^a^	0.09	0.021
	6 h	6.30	6.29	6.36	6.21	0.03	0.485
TVFA	0 h	18.76	19.05	18.94	19.24	0.25	0.942
	3 h	22.79^b^	23.70^ab^	24.19^a^	23.41^ab^	0.20	0.045
	6 h	22.59^b^	23.52^ab^	24.03^a^	23.20^ab^	0.20	0.037
NH3-N	0 h	8.12	8.25	8.35	8.53	0.31	0.980
	3 h	9.16^b^	9.75^a^	9.76^a^	9.99^a^	0.10	0.004
	6 h	8.29^b^	8.49^b^	8.99^a^	8.89^a^	0.10	0.005

Con: silage of corn (100% fresh corn without *Azolla pinnata***);** 5Ap: mixture of (95% fresh corn + 5% *Azolla pinnata*); 10Ap: mixture of (90% fresh corn + 10% *Azolla pinnata*); 15Ap: mixture of (85% fresh corn + 15% *Azolla pinnata*); TVFA: total volatile fatty acids; NH_3_-N: ammonia nitrogen

Total VFA at 3 and 6 h post-feeding were significantly higher (*P* = 0.045 and *P* = 0.037, respectively) for lambs fed 10Ap silage than control, 5Ap, and 15Ap silage, while the change in VFA concentration at 3 and 6 h post-feeding was not significant (*P* > 0.05) within groups fed corn silage mixed with different levels of Azolla, which are significantly higher (*P*˂0.05) than corn silage.

The rumen NH3-N touched its highest concentrations at 3 h post-feeding, with no significant differences between the lambs’ groups fed any of three varieties of corn silage mixed with Azolla. However, they were significantly higher (*P* = 0.004) than those in lambs fed corn silage. Thereafter, rumen NH3-N concentrations decreased slightly at 6 h post-feeding, and their concentrations were significantly higher (*P* = 0.005) in lambs fed 15Ap and 10Ap silage than those fed 5Ap and corn silage.

### Growth performance

The growth performance of experiential animals fed corn silage or corn silage mixed with different levels of Azolla is shown in Table 5. There were no significant differences in the animals’ initial and BW30 weights between the four silage varieties under study (*P* > 0.05).

**Table 5:** Growth performance of Barki male lambs fed corn silage or corn silage mixed with different levels of Azolla

Item	Experimental diets				SEM	*P* value
	Con	5Ap	10Ap	15Ap		
Initial BW, kg	31.14	31.08	31.16	31.05	0.20	0.998
BW30, kg	34.01	34.48	34.75	34.70	0.20	0.611
BW60, kg	36.98^b^	37.83^ab^	38.33^a^	38.38^a^	0.21	0.044
Final BW, kg	40.15^c^	40.91^bc^	41.75^ab^	41.96^a^	0.23	0.003
BWG, kg	9.01^c^	9.84^bc^	10.59^ab^	10.91^a^	0.23	0.005
ADG, g/day	100.1^c^	109.3^bc^	117.6^ab^	121.2^a^	2.61	0.005

Con: silage of corn (100% fresh corn without *Azolla pinnata***);** 5Ap: mixture of (95% fresh corn + 5% *Azolla pinnata*); 10Ap: mixture of (90% fresh corn + 10% *Azolla pinnata*); 15Ap: mixture of (85% fresh corn + 15% *Azolla pinnata*); BW: body weight; BWG: body weight gain; ADG: average daily gain

The BW60 of lambs fed corn silage mixed with Azolla at different levels was improved (*P* = 0.044) compared to lambs fed corn silage. The improvement in body weight was clearest (*P* = 0.003) at the end of the growth experiment (day 90). The highest BW of lambs was recorded in lambs fed 15Ap, followed by those fed 10Ap and 5Ap silage, while the lowest BW of lambs was recorded in lambs fed corn silage.

In addition, total body weight gain (BWG) and average daily gain (ADG) were significantly improved (*P* = 0.005 and *P* = 0.005, respectively) in lambs fed corn silage mixed with Azolla compared to those fed silage from corn only. The highest values of BWG and ADG were recorded in lambs fed 15Ap, followed by those fed 10Ap and 5Ap, while the lowest values were recorded in lambs fed control silage.

### Blood parameters

Table 6 shows that the hematological blood parameters of the lambs in the experimental groups were not significantly (*P* > 0.05) changed by feeding corn silage or corn silage mixed with different levels of Azolla and were within normal ranges. Furthermore, feeding corn silage or corn silage mixed with different levels of Azolla did not significantly (*P* > 0.05) alter the biochemical blood parameters of the lambs in the experimental groups, except for total protein and albumin. Lambs fed 10Ap and 15Ap had the greatest total protein and albumin values (*P* = 0.027 and *P* = 0.021, respectively), with no significant difference between them, followed by lambs fed 5Ap, while those fed corn silage had the lowest values.

**Table 6:** Blood hematological and biochemical parameters of Barki male lambs fed corn silage or corn silage mixed with different levels of Azolla

Item	Experimental diets				SEM	*P* value
	Con	5Ap	10Ap	15Ap		
Blood hematological parameters						
Hb, g/dl	11.83	11.37	12.17	11.80	0.49	0.968
RBCs, ×10^6^/µL	4.64	4.73	4.39	4.36	0.22	0.937
WBCs, µL	5080	5087	5083	5093	3.42	0.960
PLT, µL	5620.7	5629	5700.0	5704.0	135.3	0.606
MPV, fL	13.57	13.67	13.73	13.77	0.67	0.967
MCV, fL	32.70	34.03	32.90	32.63	0.26	0.197
MCH, pg	25.57	23.77	28.10	27.77	1.07	0.502
MCHC, g/dl	78.37	78.83	79.47	79.13	2.25	0.999
Blood biochemical parameters						
Total protein, g/dl	5.73^c^	5.90^bc^	6.50^a^	6.33^ab^	0.12	0.027
Albumin, g/dl	3.27^b^	3.37^b^	4.13^a^	3.73^ab^	0.12	0.021
Globulin, g/dl	2.46	2.53	2.37	2.60	0.05	0.411
Glucose, mg/dl	64.67	64.33	68.00	66.33	3.30	0.985
Urea, mg/dl	53.33	51.67	52.67	53.67	0.67	0.787
Creatinine, mg/dl	1.66	1.71	1.64	1.63	0.04	0.895
ALT, U/l	18.80	18.53	16.73	16.90	1.19	0.922
AST, U/l	126.3	120.7	123.3	128.3	1.41	0.245
ALP, U/l	188.3	189.0	189. 7	185.3	1.41	0.679

Con: silage of corn (100% fresh corn without *Azolla pinnata*); 5Ap: mixture of (95% fresh corn + 5% *Azolla pinnata*); 10Ap: mixture of (90% fresh corn + 10% *Azolla pinnata*); 15Ap: mixture of (85% fresh corn + 15% *Azolla pinnata*); Hb: hemoglobin; RBCs: red blood cell; WBCs: white blood cell; PLT: platelets; MPV: mean platelets volume; MCV: mean cell volume; MCH: mean corpuscular volume; MCHC: mean corpuscular hemoglobin concentration; ALT: alanine transaminase; AST: aspartate transaminase; ALP: alkaline phosphatase

## Discussion

### Silage evaluation

The results in Table 2 show that including Azolla at different levels improved corn silage physical properties, as both the pH and DM content decreased and the Fleig score improved. The decrease in pH and DM content in corn silage mixed with different levels of Azolla may result from higher moisture (70%) and lower DM content (30%) in the used Azolla compared to the fresh corn plants (65 and 35%, respectively). In the same vein, Li et al. (2023) found that the pH decreased significantly (*P* < 0.05) with increasing moisture content. In addition, Li et al. (2023) and Ferraretto et al. (2018) reported that high moisture content before ensiling increases lactic acid production and decreases acetic acid production as a result of the more active growth of the dominant bacterial community compared to low moisture content. It is clear from the results in Table 2 that there is an increase in lactic acid and a decrease in acetic acid levels in corn silage mixed with different levels of Azolla than in corn silage.

The chemical composition of the four varieties of silage under investigation shows that CP content was increased (*P* = 0.007) and CF content was decreased (*P* = 0.023) in corn silage mixed with different levels of Azolla than in corn silage (Table 2). Partial inclusion of Azolla instead of corn results in an increase in the CP content of the silage because Azolla has a greater CP content than corn. Previous studies considered *Azolla pinnata* a good source of high-quality protein, which can be included in non-traditional ration formulations for livestock, poultry, and aquaculture species due to its high CP content, which ranges between 18.58 and 32.8% on a dry matter basis (Al-Suwaiegh 2023; Abou El-Fadel et al. 2020; Kumar and Chander 2017).

### Nutrient digestibility

Except for EE, all nutrient digestibility coefficients were better in corn silage mixed with Azolla than in corn silage alone; the highest values were obtained in corn silage mixed with 10 and 15% of Azolla. Our obtained results agreed with those by Hassanein et al. (2023) on Zaraibi dairy goats, by Bhatt et al. (2021) on Sahiwal calves, and by Indira et al. (2009) on buffalo calves. They reported a significantly higher digestibility coefficient of DM, CP, CF, EE, and NFE in diets containing Azolla. According to Abou El-Fadel et al. (2020), adding Azolla to the diets of crossbred Osimi lambs increased the digestibility of CF and EE while decreasing those of DM, OM, CP, and NFE and the feeding value (TDN and DCP). The current study’s positive nitrogen balance is consistent with the findings of Kumar et al. (2015b), who discovered that growing Barbari goats fed a diet consisting of a 75% concentrate mixture and 25% Azolla meal retained nitrogen as a percentage of intake-N significantly higher than the control group, which was fed a diet containing a 100% concentrate mixture. Conversely, Sihag et al. (2018) revealed that retained nitrogen in growing goats is not affected by the replacement of concentrate mixture with Azolla up to 15% levels in the berseem-based total mixed ration.

High protein content and some beneficial compounds of Azolla may play an important role in improving digestibility coefficients nutritional value and nitrogen balance, which are reflected in improved body growth and health. This trend has been supported in some previous work because green Azolla contains amino acids and pro-vitamins (Hossiny et al. 2008), vitamin B12 (Leterme et al. 2010), and minerals such as calcium, potassium, phosphorus, ferrous, magnesium, and copper (El Naggar and El-Mesery 2022; Roy et al. 2016). Moreover, Anitha et al. (2016a) suggested using Azolla as an unconventional feed for livestock because it provides macronutrients such as calcium, magnesium, potassium, and vitamins such as vitamin A and B12.

### Rumen fermentation parameters

The values of pH, total VFA and NH3-N produced in the rumen of lambs fed corn silage mixed with Azolla at the three levels under study increased significantly at 3 h post-feeding. The same trend was obtained 6 h post-feeding for total VFA and NH3-N. Similar findings were noticed by Kumar et al. (2015a), who suggested that sampling time significantly affected the rumen fermentation parameters, with the highest values recorded at 4 h post-feeding in both buffalo bull groups fed Azolla or the control diet.

The increase in rumen pH value with increasing Azolla levels in the diet was also observed by Abou El-Fadel et al. (2020) on crossbred Ossimi lambs. Conversely, increasing Azolla levels in the diet decreases the rumen pH value for Zaraibi dairy goats (Hassanein et al. 2023) and Sirohi kids (Sharma et al. 2021). On the other hand, Kumar et al. (2015b) found that values of rumen pH of growing goats fed a concentrate diet were almost the same in goats fed a diet consisting of a 75% concentrate mixture and 25% Azolla meal.

Concerning NH3-N concentration, Abou El-Fadel et al. (2020) agree with the results of the current study and conclude that NH3-N concentration increased with increases in Azolla levels in the lamb diet after 3 h post-feeding, and these differences disappeared after 6 h post-feeding. Furthermore, Kumar et al. (2015b) noticed a higher concentration of rumen NH3-N in the growing goats fed 25% Azolla of their diet than in the control group, with no significant difference. Similar to our results, Hassanein et al. (2023) and Sharma et al. (2021) reported that the rumen total VFA increases with increasing Azolla supplementation levels in the diet. On the contrary, Abou El-Fadel et al. (2020) discovered that feeding lambs different levels of Azolla had no discernible effect on total VFA. Based on our observations in previous work, the differences in the effect of adding Azolla to livestock diets may be due to several factors, including the Azolla species, its nutritional value, the kind of diet (concentrated or roughage), and the productivity of the animal.

### Growth performance

The observed enhancement in the growth performance of lambs in the present study is similar to that obtained by Wadhwani et al. (2010), who found that BWG of weaned lambs of three different species (Marwari, Patanwadi, and Merino x Patanwadi) was significantly enhanced by increasing Azolla levels up to 20% of daily feed. On the contrary, Vahedi et al. (2021) reported that the BW and BWG of lambs were not affected by adding Azolla at 0, 10, and 20% levels to their diet. Furthermore, Abou El-Fadel et al. (2020) revealed that BWG and ADG of crossbred Osimi lambs decreased when replacing 0, 25 and 50% of the sunflower meal protein with the Azolla meal protein. They attributed the lack of effect or decrease in the BWG and ADG to low feed intake in Azolla-supplemented lambs.

The significant enhancement observed in the growth performance of lambs fed corn silage mixed with different levels of Azolla in the present study may be related to the enhancement in digestibility coefficients of nutrients, nitrogen retained in the body and rumen activity. Abbas and Mohanned (2023) indicated that partial replacement of soybean meal by 12% of the Azolla plant in the Iraqi Awassi lamb diet resulted in the best weight growth and feed conversion ratio, which was attributed to enhanced nutrient digestibility and rumen environment. In harmony, Roy et al. (2016) found that a partial replacement roughage-based diet with 5% dried Azolla increased the mean ADG of heifers by 20%. Previous studies have shown that replacing feed protein with Azolla protein positively affects the growth performance of animals fed diets with low protein content (as in corn silage), in contrast to diets containing concentrates rich in protein content.

### Blood parameters

The values of the hematological blood parameters were within the normal range. They did not show significant differences due to feeding lambs corn silage mixed with different levels of Azolla. This finding agrees with Singh et al. (2021) on weaned ram lambs, El-Deeb et al. (2021) on growing rabbitsAnitha et al. (2016b) on broiler rabbits. They revealed that hematological parameters like Hb, RBC, WBC, PCV, MCV, MCH, and MCHC were found within the normal range and unaffected by adding Azolla to the diet.

In the present study, the significant increase in serum total protein and albumin levels when lambs were fed the corn-azolla silage mixture may be due to the high protein content of Azolla. The rising proportion of Azolla in the silage enhances protein content in the Ap5, Ap10, and Ap15 diets by 4.9%, 13.5%, and 15.3%, respectively, leading to improved protein metabolism. Similar results regarding total protein and albumin were obtained by Singh et al. (2021), who reported that total protein and albumin were higher when ram lambs were fed a diet containing 20% ​​Azolla compared to the basic diet. In addition, Al-Suwaiegh (2023) and Hassanein et al. (2023) demonstrated that total protein in blood serum increased significantly when Azolla was added by 20% to goat diets compared to those fed a control diet. Otherwise, Abou El-Fadel et al. (2020) noticed a decrease in plasma total protein by adding Azolla to the diet of growing lambs. On the other hand, no significant differences were observed in most blood biochemical parameters by the inclusion of Azolla in the diets of lambs (Vahedi et al. 2021) and rabbits (Abdelatty et al. 2021; El-Deeb et al. 2021). The kind of diet offered to the animal may have an important impact on the variations in serum total protein with Azolla supplementation. Notably, the improvement in serum total protein occurs when adding Azolla to a diet low in protein content (such as corn silage or roughage). In contrast, Azolla does not clearly affect the total protein level in serum when added to a diet high in protein content.

## Conclusion

This study is considered the first to investigate the effect of using and including Azolla mixed with corn to make silage. The results showed improvements in the produced silage’s physical, chemical, and fermentation characteristics. Azolla (*Azolla pinnata*) can be used as a partial component of corn silage up to 15%, with beneficial effects on the nutrient digestibility, rumen fermentation, blood parameters, and growth performance of Barki lambs. We recommend that Azolla (*Azolla pinnata*) can be included in corn silage making at 10–15% in lamb feeding. More research is required to investigate the effect of different verities and levels of Azolla on the nutrient digestibility, rumen fermentation, and growth performance of Barki lambs.

## Data Availability

The data that support the findings of this study are available from the corresponding author upon reasonable request.
